# Clearance and Safety of the Radiocontrast Medium Iopamidol in Peritoneal Dialysis Patients

**DOI:** 10.4061/2011/657051

**Published:** 2011-10-19

**Authors:** Shingo Hatakeyama, Akihiko Abe, Takehiro Suzuki, Yasuhiro Hashimoto, Takuya Koie, Tomihisa Funyu, Shigeru Satoh, Tomonori Habuchi, Chikara Ohyama, Shigeki Matsuo

**Affiliations:** ^1^Department of Advanced Transplant and Regenerative Medicine, Hirosaki University Graduate School of Medicine, Hirosaki 036-8562, Japan; ^2^Department of Urology, Akita City Hospital, Akita 010-0933, Japan; ^3^Department of Urology, Hiraka General Hospital, Hiraka 013-0042, Japan; ^4^Department of Urology, Oyokyo Kidney Research Institute, Hirosaki 036-8243, Japan; ^5^Department of Urology, Hirosaki University Graduate School of Medicine, Hirosaki 036-8562, Japan; ^6^Department of Urology, Akita University Graduate School of Medicine, Akita 010-8543, Japan

## Abstract

Although the characteristics and safety of radiocontrast media in peritoneal dialysis (PD) patients are not yet well defined, their use in PD patients is considered generally safe. In this study, we evaluated clearance and adverse events of iopamidol in PD patients. We measured the iopamidol concentration in the plasma, dialysate, and urine of 11 patients. Iopamidol clearance from patient plasma was delayed with a half-life of 33.3 h, and the elimination ratio was 83.6% for 96 h. We retrospectively investigated adverse events occurring in a total of 50 stable PD patients who underwent a total of 64 angiographic computed tomography (CT) scans. In 64 angiographic CT scans, two cases of adverse events were observed. Our results suggest that iopamidol can be eliminated by regular PD and careful observation for adverse events are necessary for the safe use of radiocontrast media.

## 1. Introduction

 With the increased incidence of chronic renal failure, radiocontrast studies are important for diagnosis and treatment. Therefore, the use of iodinated radiocontrast media in computed tomography (CT) and angiography is often required in these patients [1–3]. The European Society for Urogenital Radiology guidelines, which were published in 1999, did not recommend hemodialysis (HD) after the use of iodinated radiocontrast media in continuous ambulatory peritoneal dialysis (CAPD) patients [4–6]. However, a few studies have addressed the peritoneal clearance and safety of iodinated radiocontrast media in CAPD patients [7–9]. Peritoneal dialysis (PD) is effective for removing iodinated radiocontrast media from the body, but it takes longer time than HD. The accumulation or delayed elimination of iodinated radiocontrast media may cause adverse events in chronic dialysis patients with anuria or cardiovascular dysfunction. Several reports have demonstrated acute adverse events such as heat sensations, acute urticaria, and iododerma, and delayed reactions such as vasculitis, skin disorders, submandibular swelling, and sialoadenitis even with the use of low osmolar nonionic contrast media [2, 7, 10–13]. However, not much is known about the adverse events caused by delayed elimination. In this study, we evaluated the clearance of iopamidol, a low osmolar nonionic iodinated radiocontrast media, and the incidence of adverse events associated with its use in CAPD patients.

## 2. Subjects and Methods

**Figure 1:**
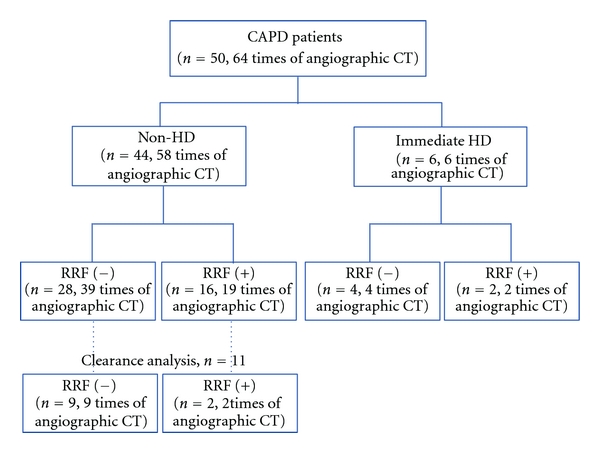
Patient classification. Forty-four patients who underwent a total of 58 angiographic CT scans were enrolled in the nonhemodialysis (non-HD) group, and six patients with a total of six angiographic CT scans were enrolled in the immediate HD group. Eleven patients in the non-HD group who participated in the clearance of iopamidol analysis.

Between 2002 and 2009, 50 patients who had undergone a total of 64 angiographic CT scans while receiving CAPD at the Akita City Hospital and the Oyokyo Kidney Research Institute were enrolled in the study (Figure 1). Patients had been stabilized on CAPD for more than 6 months at the time of the study. All patients received 1.75- to 2.75-L bags, with glucose concentrations of 1.5% or 2.5%, and four dialysate exchanges per day. CAPD treatment schedules and medications were not altered during the study. This study was conducted in accordance with the Declaration of Helsinki and was approved by the Institutional Ethical Committee at the Akita City Hospital. Informed consent was obtained from all patients.

Patient characteristics are shown in Table 1. Forty-four patients who underwent a total of 58 angiographic CT scans did not undergo HD treatment (the non-HD group), and six patients who underwent six angiographic CT scans underwent HD treatment (the immediate HD group).

### 2.1. Clearance Analysis of Iopamidol

Eleven patients were enrolled for the clearance analysis of iopamidol. All CT scans were performed for annual screening of renal cell carcinoma caused by acquired cystic kidney disease. HD was not performed in any patient after iopamidol administration. Each patient received a 50 mL/body (0.94 ± 0.21 mL/kg) intravenous bolus dose of iopamidol 300 (612.4 mg/mL) for angiographic CT. Blood samples were obtained from the 11 patients at the following times: 0.5, 1, 2, 3, 6, 12, 24, 36, 48, 72, and 96 h after iopamidol administration. Total dialysate and urine were collected every 24 h for the 4 days following administration. All samples were stored at −20°C, and the iopamidol concentrations in patient plasma, dialysate, and urine were measured.

### 2.2. Iopamidol Concentration Assay

Samples were pretreated with 0.5 mol/L of perchloric acid, and, after centrifugation, the supernatant was filtered and measured. Iopamidol concentrations in plasma, dialysate, and urine were analyzed by inductively coupled plasma atomic emission spectrometry (SPS3000, Seiko Instruments Inc., Chiba, Japan).

### 2.3. Safety Analysis

For safety analysis, we observed patients for adverse events, serum creatinine (Crea), estimated glomerular filtration rate (eGFR), and urine volume for 3 months after the angiographic CT scan.

### 2.4. Statistical Analysis

Statistical analyses were performed using SPSS (SPSS Inc, ver. 12.0, Chicago, Ill, USA) and Microsoft Excel (Microsoft Corporation, Redmond, Wash., USA) programs. All values included in the figures and text are expressed as means ± SD. Datasets were compared with paired *t* test. A *P* value of <0.05 was considered significant. We could not evaluate *P* values in Table 1 (*n* = 6) and 2 (*n* = 2), because the statistical power is insufficient to detect significant differences in small numbers of patients.

## 3. Results

**Figure 2:**
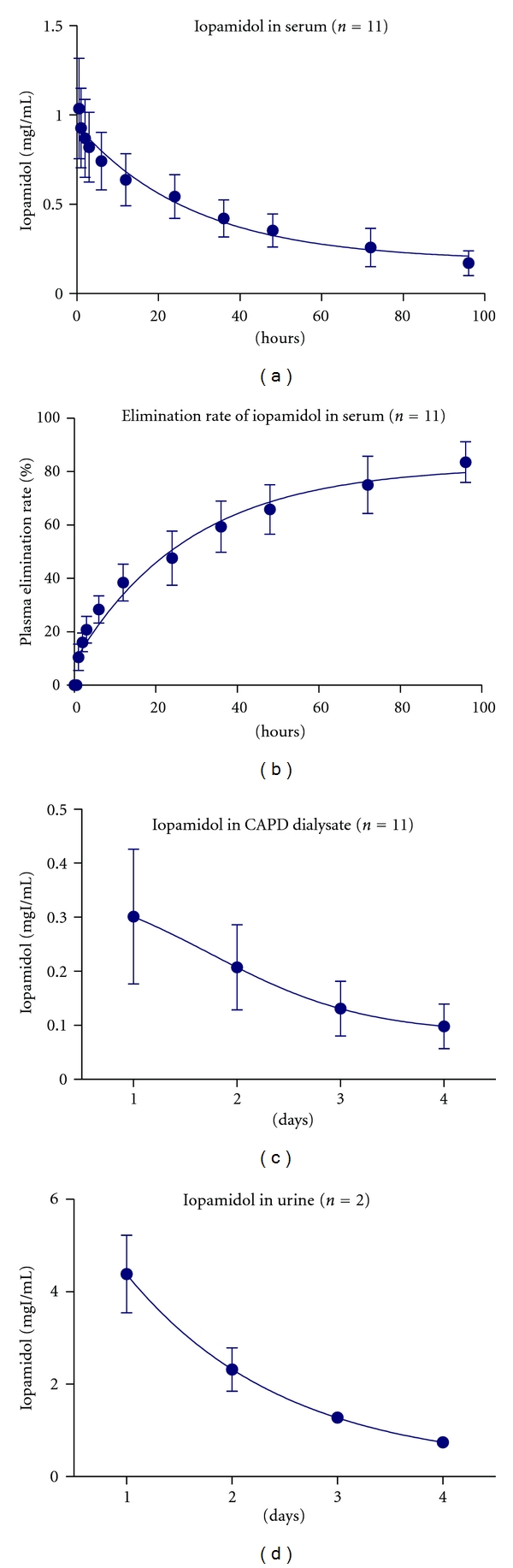
Clearance of iopamidol. Changes in iopamidol in patient plasma and clearance of iopamidol resulting from continuous ambulatory peritoneal dialysis (CAPD) are shown. Mean *T*_1/2_ of iopamidol was 33.3 ± 9.67 h (a), and the plasma iopamidol elimination ratio was 83.6%  ± 7.6% for 96 h (b). Iopamidol was eliminated from patient dialysate (c) and patient urine (d) after 4 days.

The characteristics of 11 patients in the clearance study are shown in Table 2. The mean plasma concentration versus time curve showed the mean iopamidol half-life (*T*_1/2_) of 33.3 ± 9.67 h (Figure 2(a)). The plasma iopamidol elimination ratio was 83.6%  ± 7.6% for 96 h (Figure 2(b)). 

The mean concentrations of iopamidol in dialysate and urine are shown in Figures 2(c) and 2(d). Iopamidol was removed completely from dialysate and urine. Nine patients suffered from anuria with urine volume less than 20 mL. Two patients with urine volumes of >400 mL were regarded as having residual renal function (RRF). 

**Figure 3:**
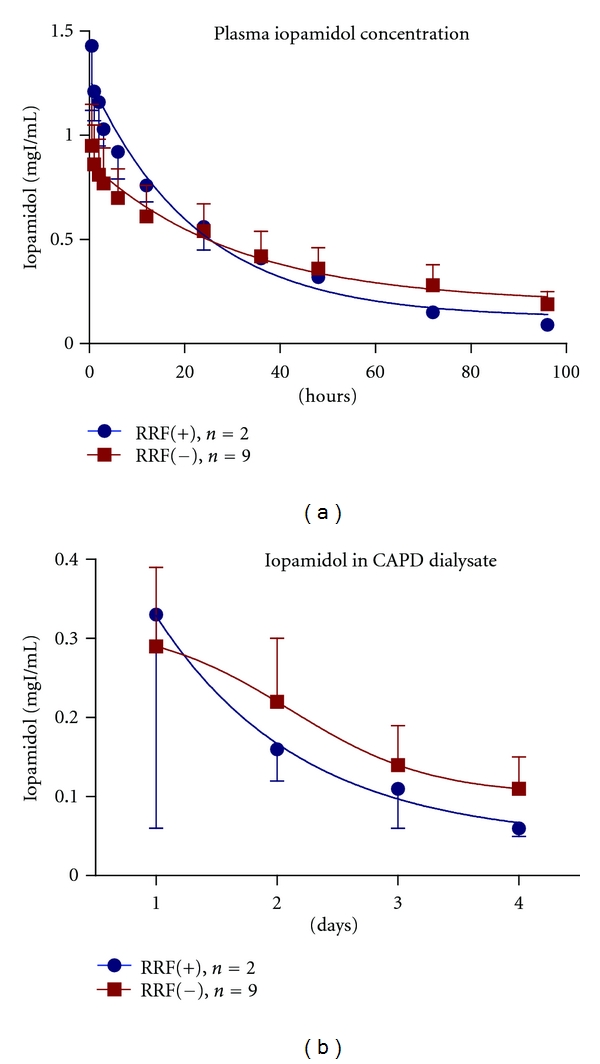
Iopamidol clearance with or without RRF. Clearance of iopamidol from the plasma of RRF (−) patients was earlier on the first day. However, the elimination rate at day 4 was significantly delayed (a). At day 4, elimination rates of RRF (+) and RRF (−) patients were 93.7%  ± 3.90% and 80.1%  ± 5.88%, respectively, and *T*_1/2_ of RRF (+) and RRF (−) patients were 21.9 ± 2.92 h and 35.8 ± 8.71 h, respectively. Dialysate iopamidol concentration in RRF (−) patients was higher than that of RRF (+) patients at day 4.

Iopamidol clearance from the plasma of patients with RRF (RRF (+)) and without RRF (RRF (−)) is illustrated in Figures 3(a) and 3(b). Clearance from RRF (−) patients was earlier on the first day, but the elimination rate at day 4 was delayed. The elimination rates of RRF (+) patients and RRF (−) patients were 93.7%  ± 3.90% and 80.1%  ± 5.88% at day 4, respectively. The clearance *T*_1/2_ of iopamidol in RRF (+) patients and RRF (−) patients were 21.9 ± 2.92 h and 35.8 ± 8.71 h, respectively. Reflecting the plasma concentration results, iopamidol concentrations in the dialysate of RRF (−) patients were higher than those in RRF (+) patients at day 4. 

Results showing the incidence of adverse events, Crea, eGFR, and urine volume after angiographic CT scans are illustrated in Table 3. In the non-HD group, no acute or delayed adverse events associated with iopamidol administration were observed in patients, regardless of whether they were classified as RRF (+) or RRF (−). On the other hand, of the six patients in the immediate HD group, two patients showed adverse events; one had a skin disorder, and the other had atrial fibrillation. There were no difference in Crea, eGFR, and urine volume after the angiographic CT for 3 months in non-HD group, but we could not evaluate statistical difference in immediate HD group because of small number of patients.

## 4. Discussion

Iopamidol is a nonionic water-soluble radiographic contrast media with low osmolarity, low chemical toxicity, and absence of ionic charge [14]. This sets it apart from other available nonionic radiocontrast media, such as iomeprol, iohexol, ioversol, iopentol, and iopromide. In our study, PD effectively removed iopamidol from the body, but iopamidol elimination from patient plasma was remarkably prolonged for more than 4 days. The clearance *T*_1/2_ and elimination rates were comparable to previous reports; one reported 37.9 h and 75.2%, and the other reported 32.6 h and 84.4% [7, 9]. Plasma iopamidol concentration and iopamidol in CAPD dialysate were lower in RRF (−) patients at first day. However, at day 4, the elimination rates and the clearance *T*_1/2_ of iopamidol in RRF (−) patients showed about 14% and 14 h delay compared with RRF (+) patients, respectively. Although we could not conclude the efficacy of RRF for the elimination of radiocontrast media because of small number of patients, these results suggest that renal excretion is an important elimination pathway even in the end stage kidney. This observation is consistent with the other recent studies in CAPD patients [9]. 

We observed no significant difference in Crea, eGFR, and urine volume after the angiographic CT for 3 months with or without RRF in non-HD group (Table 3). Although eGFR is not suitable for evaluation of renal function in dialysis patients, this suggests radiocontrast media has small effects on end-stage kidney. We could not evaluate statistical difference because of small number of patients (*n* = 6) in immediate HD group.

During the course of this study, there were no adverse events in patients in the non-HD group, but two adverse events were observed in the immediate HD group. This suggests that the risk of adverse events due to iopamidol in CAPD patients is low, indicating that the use of immediate HD does not produce any benefits. However, the mechanisms and risks of related adverse events due to delayed elimination of iopamidol are still not well known, and larger studies are necessary to address the safety. 

Donnelly et al. [7] reported a case of bilateral submandibular swelling possibly occurring 20 h after the injection of iopamidol, which resolved over the next 7 days. One study showed that conventional 4-h HD therapy immediately after the use of iodinated radiocontrast media eliminated 80% of the media and that this may reduce the possible risks associated with delayed reactions [9, 15]. However, our results suggest that immediate HD is not necessary as there were no adverse events reported in the non-HD group. Furthermore, there were negative reports about the effects of HD therapy immediately after the use of iodinated radiocontrast media because it takes several HD sessions to eliminate them completely [16, 17]. One report suggested that 3 weeks are required to eliminate iodinated radiocontrast media completely by CAPD in the regular setting [17]. Frequent dialysate exchange with very short cycles may more effectively remove it from CAPD patients' circulation [18]; however, it does not produce any benefit. This is due to slower peritoneal permeability of iodinated radiocontrast media resulting from their high molecular weight. Moreover, it is impracticable and increases the risk of hypokalemia and hypovolemia [9]. These investigations suggest a decreased necessity for scheduled HD or frequent exchange of dialysate after the use of iodinated radiocontrast media in CAPD patients because of a lower incidence ratio of adverse events. 

Because of insufficient statistical power to detect significant differences in small number of patients, our results cannot be generalized over the broader CAPD population. We believe that our observations will serve to increase understanding of the potential risks of iodinated radiocontrast media and also assist further prospective studies to further evaluate the efficacy and safety in CAPD patients.

## 5. Conclusion

Our results suggest that iopamidol can be eliminated from patients by regular CAPD schedules, and the incidence of adverse events due to iopamidol in CAPD patients is low. However, high levels of iodinated radiocontrast media in the circulation may remain for several days, and this may increase the general risk of adverse events. Adequate preparation and careful observation for adverse events are essential for the safe use of radiocontrast media.

## Figures and Tables

**Table 1:** Patient characteristics. Patients were divided into two groups: nonhemodialysis (non-HD) group and the immediate HD group. Crea: serum creatinine, eGFR: estimated glomerular filtration rate.

Patient's characteristics	All	non-HD	immediate HD
*n*	50	44	6
Angiographic CT (times)	64	58	6
Age	55.0 ± 13.1	54.9 ± 13.2	55.8 ± 9.8
Gender (M/F)	34/16	30/14	5/1
Diabetes mellitus (+/−)	11/39	11/33	0/6
Dialysis duration (M)	58.4 ± 44.1	58.6 ± 43.5	72.1 ± 52.1
Dialysate (L/day)	8.84 ± 1.54	8.90 ± 1.58	8.40 ± 0.89
Urine volume (mL/day)	117 ± 183	119 ± 186	78.1 ± 127
Crea (mg/dL)	11.2 ± 3.2	11.1 ± 3.3	11.4 ± 2.5
eGFR (mL/min/1.73m^2^)	4.50 ± 1.7	4.60 ± 1.8	4.00 ± 0.7
Iopamidol (mL/body)	82.0 ± 24.0	80.2 ± 24.7	100 ± 0.00

**Table 2:** Patient characteristics for clearance analysis of iopamidol. Patients were divided into two groups based on their residual renal function (RRF). RRF was defined by a urine volume of more than 400 mL/day. BMI: body mass index.

Clearance analysis	All	RRF (+)	RRF (−)
*n*	11	2	9
Age	62.4 ± 12.6	61.0 ± 13.6	68.5 ± 3.5
Gender (M/F)	6/5	1/1	5/4
Diabetes mellitus (+/−)	5/6	1/1	4/5
Dialysis duration (M)	57.8 ± 23.3	64.0 ± 15.6	51.4 ± 25.2
Dialysate (L/day)	8.58 ± 1.22	7.80 ± 1.19	8.76 ± 1.22
BMI	22.1 ± 3.55	21.8 ± 2.20	22.2 ± 3.89
Crea (mg/dL)	9.80 ± 2.66	7.40 ± 1.41	10.3 ± 2.61
eGFR (mL/min/1.73m^2^)	4.56 ± 1.03	5.61 ± 0.10	4.32 ± 0.99
Urine volume (mL/day)	85.0 ± 182	450 ± 70.7	3.89 ± 7.82
Iopamidol administration (mL/body)	50.0 ± 0.00	50.0 ± 0.00	50.0 ± 0.00
Plasma elimination ratio (%)*	83.6 ± 7.60	93.7 ± 3.90	80.1 ± 5.88
*T*_1/2_ (hours)	33.3 ± 9.67	21.9 ± 2.92	35.8 ± 8.71
Dialysate concentration*	0.10 ± 0.04	0.06 ± 0.01	0.11 ± 0.04

*at day 4.

**Table 3:** Adverse events and residual renal function after angiographic CT scans. The incidence of adverse events, Crea, eGFR, and urine volume for 3 months after angiographic CT scans is shown. Two patients showed adverse events within one day, in immediate HD group. No adverse event was observed in non-HD group. Crea, eGFR, and urine volume were not different before and after angiographic CT scans. Statistical analysis in non-HD group was compared to baseline, 1, 2, and 3 months (*paired t* *test*).

non-HD	RRF (+)	Crea	eGFR	Urine vol.	RRF (−)	Crea	eGFR	Urine vol.
*n*	16				28			
Angiographic CT (times)	19				39			
Adverse event	0				0			
Baseline		11.8 ± 6.4	4.97 ± 2.47	442 ± 424		11.2 ± 2.56	4.23 ± 1.00	6.77 ± 29.3
1 month		11.5 ± 4.3	5.23 ± 3.35	451 ± 387		11.1 ± 2.53	4.28 ± 0.96	11.6 ± 50.9
2 months		11.3 ± 3.7	4.85 ± 2.44	415 ± 345		11.0 ± 2.26	4.27 ± 0.88	3.71 ± 16.7
3 months		11.3 ± 3.7	4.93 ± 2.51	414 ± 360		10.9 ± 2.30	4.33 ± 0.96	8.67 ± 35.5
*P value*		*n.s.*	*n.s.*	*n.s.*		*n.s.*	*n.s.*	*n.s.*

Immediate HD	RRF (+)	Crea	eGFR	Urine vol.	RRF (−)	Crea	eGFR	Urine vol.

*n*	2				4			
Angiographic CT (times)	2				4			
Adverse event	1				1			
Baseline		11.1 ± 1.60	4.47 ± 0.35	275 ± 95.7		11.6 ± 2.90	3.83 ± 0.77	12.5 ± 28.0
1 month		12.9 ± 4.60	3.97 ± 1.15	175 ± 177		11.8 ± 3.88	3.78 ± 1.00	17.5 ± 28.7
2 months		12.2 ± 3.96	4.15 ± 1.05	150 ± 70.7		11.9 ± 2.37	3.60 ± 0.37	5.25 ± 6.08
3 months		12.5 ± 4.10	4.04 ± 1.04	300 ± 141		12.3 ± 2.09	3.45 ± 0.42	6.25 ± 7.50
